# Solitary Peutz-Jeghers type hamartomatous polyps in the duodenum are not always associated with a low risk of cancer: two case reports

**DOI:** 10.1186/1752-1947-5-240

**Published:** 2011-06-27

**Authors:** Yusuke Sekino, Masahiko Inamori, Mitsuru Hirai, Kaori Suzuki, Kaoru Tsuzawa, Keiko Akimoto, Ayako Takahata, Nobutaka Fujisawa, Kumiko Saito, Akisa Tsunemi, Michio Tanaka, Hiroshi Iida, Yasunari Sakamoto, Hirokazu Takahashi, Tomoko Koide, Chikako Tokoro, Yasunobu Abe, Atsushi Nakajima, Shin Maeda, Shigeru Koyama

**Affiliations:** 1 Gastroenterology Division, Yokohama City University School of Medicine, 3-9 Fukuura Kanazawa-ku, Yokohama, 236-0004, Japan; 2 Department of Gastroenterology, Tokyo Metropolitan Hiroo Hospital, 2-34-10 Ebisu, Shibuya-ku, Tokyo, 150-0013, Japan; 3 Department of Pathology, Tokyo Metropolitan Hiroo Hospital, 2-34-10 Ebisu, Shibuya-ku, Tokyo, 150-0013, Japan

## Abstract

**Introduction:**

A hamartomatous polyp without associated mucocutaneous pigmentation or a family history of Peutz-Jeghers Syndrome is diagnosed as a solitary Peutz-Jeghers type hamartomatous polyp. As compared with Peutz-Jeghers Syndrome, Peutz-Jeghers type hamartomatous polyps are diagnosed with a lower risk of cancer and are regarded as a different disorder.

**Case presentation:**

In case one, we describe an 84-year-old Japanese man with a 14 mm duodenal polyp. Endoscopic mucosal resection was performed and histological examination showed findings suggestive of a hamartomatous polyp with a focus of well-differentiated adenocarcinoma. In case two, we describe a 76-year-old Japanese man who had been treated for prostate, rectal and lung cancer. Upper gastrointestinal endoscopy revealed a duodenal polyp measuring 15 mm in diameter. Endoscopic mucosal resection was performed, and histological examination showed findings suggestive of a hamartomatous polyp. Liver and thyroid cancers were found after the endoscopic treatment.

**Conclusion:**

Although duodenal solitary hamartomatous polyps are associated with a lower risk of cancer, four patients, including our cases, have been diagnosed with cancerous polyps. Patients with duodenal solitary hamartomatous polyps should be treated by endoscopic or surgical resection and need whole-body screening.

## Introduction

Peutz-Jeghers Syndrome (PJS) is a rare autosomal dominant syndrome which is characterized by gastrointestinal hamartomatous polyps and mucocutaneous pigmentation [1,2], first described by Peutz in 1921 [3].

A hamartomatous polyp without associated mucocutaneous pigmentation or a family history of PJS is diagnosed as a solitary Peutz-Jeghers type hamartomatous polyp [4]. As compared with PJS, Peutz-Jeghers type hamartomatous polyps are diagnosed with a lower risk of cancer [5] and have been regarded as a different disorder from PJS.

We report two cases with a solitary Peutz-Jeghers type hamartomatous polyp treated by endoscopic mucosal resection.

## Case Presentations

**Figure 1:**
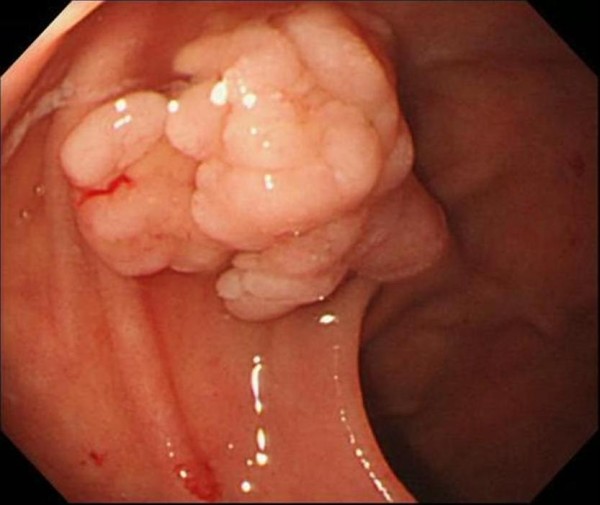
**A lobular duodenal polyp measuring 14 mm in diameter was detected in the superior duodenal angle**.

**Figure 2:**
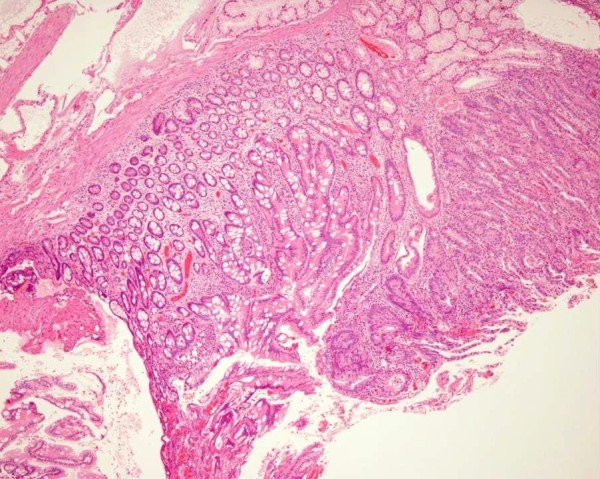
**Histological examination showed findings suggestive of a hamartoma: branching bundles of smooth muscle fibers covered by hyperplastic duodenal mucosa, with a focus of well-differentiated adenocarcinoma**. Hematoxylin and eosin staining (objective 10 ×).

Case 1 is an 84-year-old Japanese man with previous medical history of hypertension, chronic hepatitis C infection, idiopathic thrombocytopenic purpura and colon polyps (tubular adenoma and tubulovillous adenoma). He had no mucocutaneous pigmentation or family history of PJS. An upper gastrointestinal endoscopy revealed a lobular polyp measuring 14 mm in diameter, in the superior duodenal angle (Figure 1). Endoscopic mucosal resection was performed without complication and histological examination showed findings suggestive of a hamartomatous polyp--branching bundles of smooth muscle fibers covered by hyperplastic duodenal mucosa--with a focus of well-differentiated adenocarcinoma (Figure 2). A colonoscopy and small-intestinal follow-through showed no other polyps.

**Figure 3:**
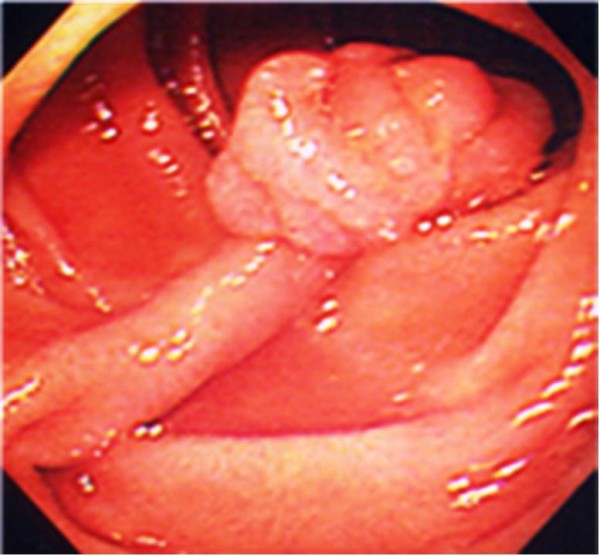
**Pedunculated duodenal polyp measuring 15 mm in diameter in the second part of the duodenum**.

**Figure 4:**
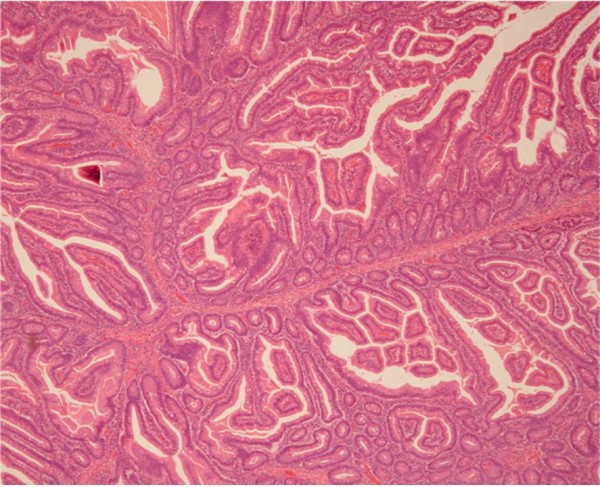
**Histological examination showed findings suggestive of a hamartoma without malignant components**. Hematoxylin and eosin staining (objective 10 ×).

Case 2 is a 76-year-old Japanese man who had been treated for prostate, rectal and lung cancer, with no mucocutaneous pigmentation or family history of PJS. An upper gastrointestinal endoscopy revealed a duodenal polyp measuring 15 mm in diameter in the second part of his duodenum (Figure 3). Endoscopic mucosal resection was performed, and histological examination showed findings suggestive of a hamartomatous polyp (Figure 4). A colonoscopy and small-intestinal follow-through showed no other polyps. After the endoscopic treatment, concomitant liver and thyroid cancers were found.

## Discussion

As compared with PJS, Peutz-Jeghers type hamartomatous polyps are diagnosed at a more advanced age, in the absence of mutation of the STK11/LKB-1 gene, and without familial history and mucocutaneous pigmentation [5].

Previous reports showed that polyps due to PJS had 3-6% of neoplastic change, such as adenomas or carcinomas [4-18]. A search of case reports on the MEDLINE database up to July 2010, using the terms "hamartomatous polyp" and "duodenum", and of reference lists of published articles (including our cases), showed 27 patients with a solitary Peutz-Jeghers type hamartomatous polyp in the duodenum (Table 1). Although solitary Peutz-Jeghers type hamartomatous polyps have been considered to show a lower potential for malignant transformation as compared to PJS, three cases (including ours) of solitary Peutz-Jeghers type hamartomatous polyps with malignant components have been reported since 2008, and the total malignant transformation rate of solitary Peutz-Jehgher type hamartomatous polyps was four out of 27 (14.8%). There were no significant tendencies of malignant transformation within the age or sex of the patient, or the location, size or endoscopic appearances of the polyp.

**Table 1:** Twenty-seven cases of solitary duodenal Peutz-Jeghers type hamartomatous polyps.

Author	Year	Number of patients	Age	Sex	Location	Surface	Size (mm)	Treatment	Malignant transformation
Gannon [6]	1962	6	NS	NS	NS	Smooth	NS	NS	No
Shiegel [7]	1978	1	75	NS	2nd	Smooth	NS	surgery	No
Ushijima [8]	1986	1	46	M	2nd	Lobulated	20 × 20 × 15	endoscopy	No
Bott [9]	1986	1	23	M	4th	NS	50 × 40	surgery	No
Naitoh [10]	1988	1	56	F	3rd	Smooth	30 × 15	endoscopy	No
Rossetti [11]	1989	1	22	F	2nd	Smooth	50	endoscopy	No
Tanaka [12]	1990	2	41	M	3rd	Lobulated	25 × 18	endoscopy	No
			82	F	2nd	Lobulated	25 × 20	endoscopy	No
Nebri [4]	1993	1	63	F	1st	NS	50 × 35	surgery	No
Ichiyoshi [13]	1996	1	84	F	2nd	Lobulated	25 × 20	endoscopy	Yes
Oncel [14]	2003	2	68	F	3rd	NS	15	endoscopy	No
			53	M	2nd	Multiple polyps	5	endoscopy	No
Kitaoka [5]	2004	1	22	F	1st	Lobulated	35 × 30 × 30	endoscopy	No
Itaba [15]	2006	2	87	F	2nd	NS	17	endoscopy	No
			56	M	1st	Lobulated	12	endoscopy	No
Suzuki [16]	2008	3	59	F	2nd	Lobulated	15 × 15	surgery	No
			68	F	2nd	Lobulated	10 × 8	endoscopy	Yes
			60	F	1st	Lobulated	10 × 10	endoscopy	No
Jamaludin [17]	2009	1	46	M	1st	Lobulated	70 × 40	surgery	Yes
Kantarcioglu [18]	2009	1	28	M	2nd	Lobulated	25 × 15	endoscopy	No
Sekino: our report	2010	2	84	M	2nd	Lobulated	16 × 13	endoscopy	Yes
			76	M	2nd	Lobulated	15	endoscopy	No

NS: not stated Ethnicity

The most serious problem in PJS is an increased risk of cancer in the gastrointestinal tract. The occurrence of cancer in the gastrointestinal tract has been reported in 20-25% of patients with PJS, and a risk of cancer in other organs has been also reported, including the ovary, breast, bladder, pancreas and thyroid [2,19-22].

To the best of our knowledge, there have been no previous reports of patients with solitary Peutz-Jeghers type hamartomatous polyps presenting with malignancy in other organs. This is one of the reasons that solitary Peutz-Jeghers type hamartomatous polyps have been considered as a separate clinical entity from PJS. However, Case 2 in our report had duplicated malignancy in six organs. An overlap between solitary Peutz-Jeghers type hamartomatous polyps and PJS may need to be re-examined.

Our two cases were diagnosed in patients with an advanced age similar to previous reports, but they differ in the malignant alteration of a hamartomatous polyp and concomitant other cancers. Patients with duodenal Peutz-Jeghers type hamartomatous polyps should undergo colonoscopy and whole-body screening; duodenal solitary Peutz-Jeghers type hamartomatous polyps should preferably be treated by endoscopic or surgical resection.

## Conclusions

We report two cases of duodenal solitary Peutz-Jeghers type hamartomatous polyp. Case 1 was a hamartomatous polyp with a focus of well-differentiated adenocarcinoma, and Case 2 was a hamartomatous polyp with five cancers in other organs. We advise that patients with duodenal solitary Peutz-Jeghers type hamartomatous polyps should preferably be treated with endscopic or surgical resection and whole-body screening.

## Abbreviations

PJS: Peutz-Jeghers Syndrome.

## Consent

Written informed consent was obtained from both patients for publication of these case reports and any accompanying images. Copies of the written consent are available for review by the Editor-in-Chief of this journal.

## Competing interests

The authors declare that they have no competing interests.

## Authors' contributions

YS, MI, MH, KS, KT and KA analyzed and interpreted the patient data. AT, NF, KS, AT, MT and HI analyzed endoscopic data. YS, HT, TK, CT, YA, AN and SM performed the histological examination of the organs. YS, MI and SK were major contributors in writing the manuscript. All authors read and approved the final manuscript.
